# In-Depth Analysis of Genetic Variation Associated with Severe West Nile Viral Disease

**DOI:** 10.3390/vaccines8040744

**Published:** 2020-12-08

**Authors:** Megan E. Cahill, Mark Loeb, Andrew T. Dewan, Ruth R. Montgomery

**Affiliations:** 1 Center for Perinatal, Pediatric and Environmental Epidemiology, Department of Chronic Disease Epidemiology, Yale School of Public Health, 1 Church Street, New Haven, CT 06510, USA; megan.cahill@yale.edu (M.E.C.); andrew.dewan@yale.edu (A.T.D.); 2 3208 Michael DeGroote Centre for Learning & Discovery, Division of Clinical Pathology, McMaster University, Hamilton, ON L8S 4L8, Canada; loebm@mcmaster.ca; 3 Department of Internal Medicine, Yale School of Medicine, 300 Cedar Street, New Haven, CT 06520, USA

**Keywords:** West Nile virus, West Nile neuroinvasive disease, genome-wide association study, gene-gene interactions, disease severity

## Abstract

West Nile virus (WNV) is a mosquito-borne virus which causes symptomatic disease in a minority of infected humans. To identify novel genetic variants associated with severe disease, we utilized data from an existing case-control study of WNV and included population controls for an expanded analysis. We conducted imputation and gene-gene interaction analysis in the largest and most comprehensive genetic study conducted to date for West Nile neuroinvasive disease (WNND). Within the imputed West Nile virus dataset (severe cases n = 381 and asymptomatic/mild controls = 441), we found novel loci within the MCF.2 Cell Line Derived Transforming Sequence Like (*MCF2L*) gene (rs9549655 and rs2297192) through the individual loci analyses, although none reached statistical significance. Incorporating population controls from the Wisconsin Longitudinal Study on Aging (n = 9012) did not identify additional novel variants, a possible reflection of the cohort’s inclusion of individuals who could develop mild or severe WNV disease upon infection. Many of the top gene-gene interaction results were intergenic, with currently undefined biological roles, highlighting the need for further investigation into these regions and other identified gene targets in severe WNND. Further studies including larger sample sizes and more diverse populations reflective of those at risk are needed to fully understand the genetic architecture of severe WNDD and provide guidance on viable targets for therapeutic and vaccine development.

## 1. Introduction

Mosquito-borne viruses such as West Nile virus (WNV) are a major global health threat, with estimates of more than 340 million infections and more than 440,000 fatalities each year [1]. WNV was first isolated in an individual from the West Nile region of Uganda and is now found in more than 80 countries [2,3]. Since its introduction to the United States in 1999, the virus has since spread throughout the continental states and into Mexico and Canada [4]. Among infected humans, the majority—especially the young—remain asymptomatic, while approximately 20% mainly older individuals present mild symptoms such as fever, and <1% exhibit severe, potentially life-threatening West Nile neuroinvasive disease (WNND) [5,6,7,8,9].

Genetic factors are among the host characteristics identified to place a minority of individuals at increased risk of severe disease. To date, 13 candidate-gene studies and one larger-scale 13,371 single-nucleotide polymorphisms (SNPs) study have been conducted to examine genetic factors associated with severe WNND [10]. The largest study conducted to date included 560 neuroinvasive case patients and 950 control patients genotyped for 13,371 SNPs, and identified SNPs within the genes replication factor C1 (*RFC1*), sodium channel neuronal type I α subunit (*SCN1A*), and ananyl aminopeptidase (*ANPEP*) to be associated with severe disease, with *p*-values not reaching statistical significance [11]. These genes function in several distinct cellular physiology processes with potential relevance to WNV infection and disease progression [11], but did not reveal common immune-related targets as has been noted in genome-wide association studies (GWAS) of related viral infections [12,13].

GWAS focus on common variants, most of which are associated with small increases in risk [14]. Additional risk factors for severe disease remain undiscovered, and strategies to further define risk include conduction of larger studies that better account for the diverse affected populations and through conduction of novel analyses of previous studies. Novel analyses can include incorporating population controls to increase power or assessing gene-gene or gene-environment interactions, rare variants, epigenetics, and pathways. Gene-gene interactions, or epistasis, measure the effect of variation at two distinct loci and reflect how genes and their products interact within an organism and, in this scenario, together affect disease severity. Using the first WNND GWAS published to date, we used imputation, population controls, and gene-gene interaction analysis to further identify genetic targets and better define genetic architecture for severe WNND, with the ultimate objective of providing guidance for therapeutic and vaccine development [15].

## 2. Materials and Methods

### 2.1. WNV-Infected Study Population

Study participants were enrolled between 2004 and 2009 from Nebraska, Pennsylvania, Texas, and Colorado in the United States and Alberta and Saskatchewan in Canada, as previously described [11]. All study participants were adults ≥18 years with confirmed infection with WNV based on the guidelines of the Centers for Disease Control and Prevention. The 381 cases were diagnosed with meningitis, encephalitis, or acute flaccid paralysis, while the 441 control patients did not have any of these diagnoses. Blood samples from adults were collected for DNA extraction and genotyping with the Illumina 1 Million Duo and Illumina OmniExpress as previously described (Illumina, San Diego, CA, USA) [11].

### 2.2. Quality Control and Imputation

For quality control, we used PLINK (Harvard University, Boston, MA, USA) to remove all individuals and SNPs with >5% missing rates and SNPs that deviated significantly from Hardy Weinberg Equilibrium (*p* < 1 × 10^−5^) [16]. We updated the data for human genome build 37 (hg19) using Batch Coordinate Conversion (LiftOver) [17]. Genotype phasing was conducted with SHAPEIT (University of Oxford, Oxford, UK) and imputation was performed using IMPUTE2 (University of Oxford, Oxford, UK), with 1000 Genomes Project, Phase 3 dataset serving as reference [18,19,20]. Imputation quality is scored by the INFO score (0–1.0, with a higher score indicating increased certainty), and SNPs with INFO scores >0.7 were included in the analysis. Principal component analysis was conducted on the directly genotype data using EIGENSTRAT (Harvard University, Boston, MA, USA) [21,22]. We merged the two imputed chip datasets in PLINK and subsequently conducted association analyses, with the top 10 principal components, sex, and the type of chip (1 Million Duo or OmniExpress) as covariates (Supplemental Figure S1). To adjust for multiple testing, the genome-wide Bonferroni-corrected threshold of 5 × 10^−8^ was used to assess *p*-value significance [23,24]. Manhattan and quantile-quantile (QQ) plots were created through the R package qqman [25].

### 2.3. Population Controls

The cohort from the Wisconsin Longitudinal Study on Aging (dbGaP accession: phs001157.v1.p1) was used as a population control dataset during an expanded analysis of the imputed WNV dataset [26,27]. The Wisconsin dataset was procured from dbGaP under an approved agreement [28]. As previously described, 9012 individuals were enrolled and provided samples that were genotyped on the Illumina HumanOmniExpress chip, human genome build 37 (Illumina, San Diego, CA, USA) [27,29]. Similar to the methods we used in the WNV dataset, they conducted imputation using the IMPUTE2 software (University of Oxford, Oxford, UK), with the 1000 Genomes Project, Phase 3 dataset serving as controls [20,30]. We merged this Wisconsin population controls imputed dataset with the WNV imputed dataset in PLINK, and conducted univariate analysis using the top 10 principal components, sex, the type of chip for the WNV participants (1 Million Duo or OmniExpress), and the cohort (Wisconsin population control or WNV sample) as covariates. Principal component analysis of the merged, directly genotyped datasets was conducted using EIGENSTRAT [21,22,31]. To reduce heterogeneity between the samples, we limited analysis to SNPs with population control minor allele frequencies (MAF) ±5% of the WNV control MAF.

### 2.4. Analysis of Gene-Gene Interactions

To assess the directly genotyped dataset for gene-gene interactions, we applied PLINK’s fast epistasis function, which compares genotype count tables, to test interactions between all SNPs with MAF ≥10%. We then applied the regular epistasis function, which employs a logistic regression approach, on all interactions with *p*-values below 5 × 10^−8^ from the fast epistasis test [16]. We used a Bonferroni-corrected threshold of 5 × 10^−14^ to assess *p*-value significance.

## 3. Results

### 3.1. Imputation Increases Identification of Genes Associated with WNV

To identify additional gene loci with a role in infection with WNV, we made use of imputation to analyze the existing GWAS dataset. The initial analysis covered 1,426,538 directly genotyped SNPs, and with imputation, we increased the number of loci which can be assessed for a role in WNV infection by 8-fold. Following imputation, 11,883,872 SNPs passed quality control (Supplemental Table S1). The top hits from the imputed data set revealed several novel targets including rs3065938 in an intergenic region of Chromosome 4 (*p*-value = 9.45 × 10^−7^) and rs78660320 in the Testis Expressed 15, Meiosis and Synapsis Associated (*TEX15*) gene on Chromosome 8 (1.76 × 10^−6^) (Table 1; Manhattan plot, Supplemental Figure S2; QQ plot, Supplemental Figure S3). While still short of the statistical significance threshold of 5 × 10^−8^, these imputed SNPs were more significantly associated with disease than the top associations from the original analysis (Supplemental Table S2). Other novel SNPs of potential interest that approached significance were in the MCF.2 Cell Line Derived Transforming Sequence Like (*MCF2L*) gene (rs9549655 and rs2297192; *p*-values 5.84 × 10^−7^ and 1.35 × 10^−6^), the TIMELESS Interacting Protein (*TIPIN*) gene (rs36061431; *p*-value 2.43 × 10^−6^), and the Potassium Calcium-Activated Channel Subfamily N Member 3 (*KCNN3*) gene (rs7553671; *p*-value of 2.49 × 10^−6^).

### 3.2. Population Controls Extend Univariate Analysis

The WNV dataset includes WNV patients of differing severity of disease but does not include asymptomatic subjects who make up the majority of infections [11,32]. For additional power to discern genes with a significant role in WNV infection, we added a large dataset of population controls from the Wisconsin Longitudinal Study on Aging (n = 9012) [27]. We re-analyzed the imputed WNV dataset after incorporating the imputed dataset of population controls. In order to reduce heterogeneity between samples, results were restricted to SNPs with population controls MAF within 5% of the WNV controls MAF. From the reanalysis with population controls, the top SNP was in the *MCF2L* gene (rs2297192) on Chromosome 13; *p*-value of 2.30 × 10^−6^ (Table 2).

The top SNPs from the univariate WNV-only analysis (Table 1) were not included in this analysis as the MAF between the WNV controls and Wisconsin population controls was greater than the 5% cutoff. Many of the *p*-values of the population controls analysis were similar or even slightly higher than the WNV-only data analysis, suggesting the frequency of the risk alleles may be greater in the population controls than in the WNV controls. Notably, for some of the SNPs, the population control MAF was between the WNV case MAF and WNV control MAF, limiting the potential boost in *p*-values despite the comparatively large number of population controls. No additional novel areas of interest were noted in the Manhattan plot (Supplemental Figure S4; QQ plot, Supplemental Figure S5).

### 3.3. Gene-Gene Interactions Identify Combined Effects of Variation at Two Loci of Interest

To identify potential interactions of two genes which may contribute to genetic susceptibility to infection with WNV, we examined gene-gene interactions across the genome. None of the interactions passed the Bonferroni-corrected *p*-value of 5 × 10^−14^ (Table 3). Although not reaching significance, the top interaction was between rs7021419, an intergenic SNP on chromosome 9, and rs9989408, a SNP located in the Heparan Sulfate-Glucosamine 3-Sulfotransferase 4 (*HS3ST4*) gene on chromosome 16 (*p*-value of 2.42 × 10^−11^). Other top interactions included SNPs from the Neuregulin 1 (*NRG1*), Microtubule-Associated Serine/Threonine-Protein Kinase 4 (*MAST4*), Ribosomal Protein L17 (*RPL17*) genes. No additional interactions were identified in the combined cohort of WNV cases and controls and the Wisconsin population controls. As expected, and consistent with the univariate analysis outlined above, the addition of the population controls resulted in less significant findings of gene-gene interactions.

## 4. Conclusions

With only a minority of individuals infected with WNV developing severe, potentially life-threatening disease, it is vital to identify factors associated with susceptibility to disease [33,34]. Human genetic variation is of critical importance to an individual’s response to infection and subsequent progression to severe disease and previous studies have identified several genes associated with WNV infection [35,36,37,38,39,40]. To identify additional novel genetic variants associated with severe WNND, we conducted advanced genetic analyses of loci in a North American cohort by using an imputation approach, incorporating population controls, and analyzing gene-gene interactions. Through univariate analysis of the imputed dataset, we found novel loci, particularly near the *MCF2L* gene on Chromosome 13, that may be associated with severe WNND. *MCF2L* belongs to the Rho guanine nucleotide exchange factors, which are one of three groups of proteins that control Rho GTPases [41]. Viruses have been shown to interact with and evade Rho GTPase signaling, including WNV during its infiltration of murine neurons [42,43]. While many of the other top SNPs were intergenic, one SNP is located in the *TIPIN* gene. The *TIPIN* gene product is the TIMELESS-interacting protein, which has been implicated in Epstein–Barr viral replication and at the pathway level during infection with zika, a related flavivirus [44,45,46]. These newly identified SNPs, while not reaching statistical significance in our study sample, are more significant than the findings of the original study. The advanced analysis using imputation provided important additional clues of genetic susceptibility. Future investigations, both of these and other SNPs analyzed within larger samples sizes with greater statistical power, integrated with profiles of WNV immune responses and animal models, can illuminate the potential roles of these genes on disease pathogenesis [47,48,49,50,51].

To expand the sample size and increase power for our analysis, we utilized population controls, also of northern-European descent, from the Wisconsin Longitudinal Study on Aging. We selected the control dataset due to its similarities to the WNV dataset, namely in location and subject population of individuals of northern European descent. Including the control dataset of >9000 individuals resulted in surprisingly limited improvement across SNP *p*-values. This may be due to occult infection within the Wisconsin group, or differences across MAFs, even following the removal of SNPs with population control MAFs greater than 5% different from the WNV control MAF. As shown in Table 2, the MAF in the population control dataset often fell between the MAF estimates for the WNV cases and controls, indicating the population controls represent both potential severe cases as well as asymptomatic or mild controls upon infection with WNV. The population controls are more likely to carry the risk alleles for severe disease compared to the WNV controls, a population infected with WNV but resistant to severe disease. The inclusion of an additional, external dataset could introduce potential bias through misclassification of severe cases and batch effects and confounding through population substructure [52].

Including population controls did not lead to more significant findings as anticipated, and the greatest increase in statistical power will be gained through the inclusion of a larger sample of infected cases. Total cases of WNV reported in the USA since 1999 are 50,830 [53], thus, only limited enrollment is possible. Most of the WNV genetic studies conducted to date have been relatively small in sample size, with 5 of the 13 published genetic studies including fewer than 50 severe cases [10]. We conducted our analyses in the largest WNV genetic study conducted to date, by both number of genetic variants tested and number of severe cases. However, adequate power (>80%) to identify gene-gene interactions required odds ratios of 2.4 or higher, depending on the minor allele frequencies (Supplemental Table S3). Furthermore, ongoing and future studies need to be conducted in the appropriate at-risk populations; a mutation that plays an important role in disease progression in one population may not be universally significant. WNV has been found on every continent excluding Antarctica, but published studies exploring the role of genetic variation in WNV disease severity have focused largely on populations of Northern European descent [54]. Only three of the 13 studies were conducted outside North America, and focused on Greece, Macedonia, and Israel.

When we analyzed gene-gene interactions, many of the top pairs included an intergenic region, highlighting the importance of ongoing efforts to understand the roles and effects of these areas. The top interaction involved a SNP in the *HS3ST4* gene, which encodes the enzyme heparan sulfate *D*-glucosaminyl 3-*O*-sulfotransferase 4 and has been linked to herpes simplex virus type 1 pathogenesis and tumor growth [55,56]. *HS3ST4* has been shown to be regulated by TRF2, with abnormal expression limiting natural killer cell recruitment and activation; notably, natural killer cells play an important role in the immune system response to WNV infection [57,58,59]. While the top interactions approached but did not pass the Bonferroni-corrected *p*-value threshold, future research may reveal the potential role of *HS3ST4* and other identified genes during WNV infection.

There are several potential limitations to our study design. LiftOver was used to update the genetic data to human genome build 37, which was a necessary step for the software and reference panels used in the subsequent analytical steps. While studies evaluating LiftOver and other similar conversion tools have shown low rates of errors when converting between builds [60,61], this could have potentially introduced error. To impute the genetic data for the WNV study participants, we used the IMPUTE2 software in order to match the approach previously used on the Wisconsin Longitudinal Study on Aging cohort; studies have shown similar accuracy across IMPUTE2 and other imputation software [62,63]. As imputation estimates ungenotyped loci, we only included imputed variants with an INFO quality score above 0.7 to limit potential error. In the univariate analyses, we used the Bonferroni-corrected genome-wide threshold of 5 × 10^−8^ to assess *p*-value significance. The Bonferroni-corrected threshold is widely acknowledged to be more conservative than alternative approaches [64,65], but is less likely to provide false positive results [66,67]. Our top findings, while not statistically significant using the Bonferroni-corrected threshold, are biologically plausible and may reflect the underlying biology of WNND; however, further genetic studies are needed to replicate these findings. Our analyses utilized data from the largest WNV GWAS conducted to date, but as noted above, future studies incorporating more cases and diverse populations are needed to best understand the genetic architecture of WNND. If our findings are replicated in additional cohorts, biological experiments will be needed to elucidate the functional roles of the identified genetic variation in WNV infection. The importance of understanding genetic architecture of complex traits to guide vaccine development has been well documented in other diseases [68,69,70], and our analyses show further studies need to be conducted to adequately inform and guide vaccine development.

As WNV and other mosquito-borne viruses become of increasing concern due to climate change, there is a time-sensitive need for future work to explore the roles of identified genes in disease pathogenesis and a continued need to conduct larger, more diverse studies for a more universal understanding of genetic risk factors and viable therapeutic targets. As noted here, novel analyses such as imputation, population controls, and gene-gene interactions in existing datasets may provide additional leads for future investigation, but well-designed, fully-powered new studies with sufficient asymptomatic, mild, and severe infection subgroups are needed to completely understand the genetic architecture of severe WNND [71]. In addition to guidance for much-needed vaccines and treatment, this knowledge could identify individuals at increased risk for severe disease, allowing for more targeted precaution measures and earlier intervention.

## Figures and Tables

**Table 1:** Top single-nucleotide polymorphisms (SNPs) following individual variant analysis of the imputed West Nile virus (WNV) dataset. The table shows the top 10 results by chromosome (Chr), single-nucleotide polymorphism (SNP), corresponding gene, base position, odds ratio (OR), and *p*-value. None of the interactions passed the Bonferroni-corrected *p*-value of 5 × 10^−8^. Gene acronyms: MCF.2 Cell Line Derived Transforming Sequence Like (*MCF2L*), Testis Expressed 15, Meiosis and Synapsis Associated (*TEX15*), TIMELESS Interacting Protein (*TIPIN*), Potassium Calcium-Activated Channel Subfamily N Member 3 (*KCNN3*).

Chr	SNP	Gene	Base Position	OR	*p*-Value
13	rs9549655	*MCF2L*	113725367	2.38	5.84 × 10^−7^
4	rs3065938	Intergenic	125374129	0.36	9.45 × 10^−7^
13	rs2297192	*MCF2L*	113720476	2.23	1.35 × 10^−6^
8	rs78660320	*TEX15*	30739132	0.44	1.76 × 10^−6^
13	rs701542	Intergenic	103581620	2.30	2.05 × 10^−6^
15	rs36061431	*TIPIN*	66669445	2.27	2.43 × 10^−6^
1	rs7553671	*KCNN3*	154737236	0.48	2.49 × 10^−6^
6	rs4706217	Intergenic	85705226	0.48	2.79 × 10^−6^
13	rs783448	Intergenic	103581492	2.29	2.89 × 10^−6^
13	rs783449	Intergenic	103581457	2.15	3.92 × 10^−6^

**Table 2:** Top SNPs following individual variant analysis of the imputed datasets of WNV severe cases, non-severe WNV-infected controls, and population controls from the Wisconsin Longitudinal Study on Aging. The table shows the top 10 results by chromosome (Chr), single-nucleotide polymorphism (SNP), odds ratio (OR), *p*-value from the combined WNV cohort and population control analysis, *p*-value from the WNV-only cohort analysis, and minor allele frequency (MAF) within the WNV severe cases, WNV-infected non-severe controls, and population controls from the Wisconsin Longitudinal Study on Aging. None of the interactions passed the Bonferroni-corrected *p*-value of 5 × 10^−8^. Gene acronyms: MCF.2 Cell Line Derived Transforming Sequence Like (*MCF2L*), Nicotinamide *N*-Methyltransferase (*NNMT*), Coagulation Factor X (*F10*).

Chr	SNP	Gene	OR	*p*-Value from Population Control Analysis	*p*-Value from WNV-Only Analysis	WNV Cases MAF	WNV Controls MAF	Population Controls MAF
13	rs2297192	*MCF2L*	2.13	2.30 × 10^−6^	1.35 × 10^−6^	0.203	0.127	0.167
3	rs9834139	Intergenic	1.91	1.13 × 10^−5^	5.60 × 10^−5^	0.381	0.288	0.337
1	rs1245487	LOC105378861	1.84	3.48 × 10^−5^	2.28 × 10^−5^	0.357	0.291	0.317
12	rs10850475	Intergenic	0.54	5.74 × 10^−5^	1.10 × 10^−4^	0.144	0.217	0.191
1	rs10489329	Intergenic	1.78	8.06 × 10^−5^	3.44 × 10^−4^	0.327	0.225	0.257
13	rs3814264	*MCF2L*	1.79	9.14 × 10^−5^	2.51 × 10^−5^	0.249	0.179	0.224
11	rs576598	Intergenic	0.56	9.23 × 10^−5^	4.88 × 10^−5^	0.302	0.379	0.341
11	rs694539	*NNMT*	0.54	9.71 × 10^−5^	1.47 × 10^−4^	0.133	0.206	0.183
21	rs2407581	LOC105372745	1.77	1.05 × 10^−4^	5.16 × 10^−5^	0.298	0.229	0.267
13	rs556694	*F10*	0.48	1.08 × 10^−4^	4.93 × 10^−4^	0.073	0.137	0.106

**Table 3:** Top gene-gene interactions, with corresponding chromosome (Chr), single-nucleotide polymorphism (SNP), base position (BP), and gene in the WNV dataset. None of the interactions passed the Bonferroni-corrected *p*-value of 5 × 10^−14^. Gene acronyms, in order of appearance: Heparan Sulfate-Glucosamine 3-Sulfotransferase 4 (*HS3ST4*), Neuregulin 1 (*NRG1*), Microtubule-Associated Serine/Threonine Kinase Family Member 4 (*MAST4*), Ribosomal Protein L17 (*RPL17*), Polypeptide *N*-Acetylgalactosaminyltransferase 10 (*GALNT10*), Olfactory Receptor Family 5 Subfamily R Member 1 (*OR5R1*), Solute Carrier Family 25 Member 21 (SLC25A21), CUB And Sushi Multiple Domains 1 (*CSMD1*), Forkhead Box P1 (*FOXP1*), Proline Rich 5 Like (*PRR5L*), Musashi RNA Binding Protein 2 (*MSI2*), Cadherin 13 (*CDH13*), Amphiphysin (*AMPH*).

First SNP				Second SNP				*p*-Value
Chr	SNP	BP	GENE	CH	SNP	BP	GENE	
9	rs7021419	2322572	Intergenic	16	rs9989408	25879109	*HS3ST4*	2.42 × 10^−11^
9	rs7021419	2322572	Intergenic	16	rs12708686	25881959	*HS3ST4*	6.22 × 10^−11^
3	rs941304	74887781	LOC 105377167	8	rs3847131	32282019	*NRG1*	2.08 × 10^−10^
5	rs465962	66197511	*MAST4*	18	rs1943676	47017820	*RPL17*	3.41 × 10^−10^
5	rs153411	153753481	*GALNT10*	11	rs11228306	56184659	*OR5R1*	4.04 × 10^−10^
3	rs2030874	5547942	Intergenic	14	rs17105314	37257671	*SLC25A21*	4.44 × 10^−10^
8	rs2627359	3743017	*CSMD1*	12	rs12304940	68457881	LOC 107984526	4.53 × 10^−10^
5	rs154620	66200248	*MAST4*	18	rs1943676	47017820	*RPL17*	4.57 × 10^−10^
2	rs4426483	155807051	LOC 105373696	3	rs2568844	71288864	*FOXP1*	5.04 × 10^−10^
11	rs1425936	36399514	*PRR5L*	17	rs758142	55740786	*MSI2*	6.77 × 10^−10^
16	rs173839	83782302	*CDH13*	22	rs2075120	17326432	Intergenic	7.40 × 10^−10^
7	rs10464364	38652088	*AMPH*	18	rs4468704	37727465	Intergenic	8.29 × 10^−10^

## Data Availability

Publicly available datasets were analyzed in this study. The West Nile virus dataset is available through the ImmPort Portal of National Institute of Allergy and Infectious Diseases (study accession: SDY20; doi:10.21430/M3H2LBOI83). The Wisconsin Longitudinal Study on Aging dataset is available upon application through dbGaP of National Center for Biotechnology Information (study accession: phs001157.v1.p1) [72].

## Supplementary Materials

The following are available online at https://www.mdpi.com/2076-393X/8/4/744/s1. Figure S1: Quality control and analysis flowchart. Overview of approach for quality control, phasing, imputation, and association analysis of the genetic dataset of WNV sample, Figure S2: Manhattan plot of imputed dataset of WNV severe cases and mild and asymptomatic infection controls. The chromosomes are along the x-axis and the negative log *p*-value along the y-axis. The blue line indicates SNPs that approach statistical significance, and the red line separate SNPs that reach the genome-wide corrected *p*-value threshold. The top association hits are located in a region of chromosome 13, Figure S3: QQ plot of the expected and observed negative log of the *p*-values from the association analysis of the imputed WNV dataset. The negative log of the observed *p*-values (x-axis) are plotted against the negative-log of the expected *p*-values (y-axis). The majority of the SNPs fall along the red line, indicating the results are not inflate, Figure S4: Manhattan plot of imputed datasets of WNV severe cases, non-severe WNV-infected controls, and population controls from the Wisconsin Longitudinal Study on Aging. The chromosomes are along the x-axis and the negative log *p*-value along the y-axis. The blue line indicates SNPs that approach statistical significance, with none of the SNPs reaching statistical significance, Figure S5: QQ plot of the expected and observed negative log of the *p*-values from the association analysis of the imputed datasets of WNV severe cases, non-severe WNV-infected controls, and population controls from the Wisconsin Longitudinal Study on Aging. The negative log of the observed *p*-values (x-axis) are plotted against the negative-log of the expected *p*-values (y-axis), Table S1: Imputation of the West Nile virus dataset. The number of directly genotyped SNPs and the total number of SNPs following imputation, by chromosome. The ‘before imputation’ numbers only include directly genotyped SNPs; the ‘after imputation’ includes both directly genotyped and imputed SNPs, Table S2: Comparison of *p*-values for the top three SNPs from the previously published GWAS within the current subset, with no covariate adjustment to reflect the original analysis. The previously published GWAS, including 560 neuroinvasive case patients and 950 control patients analyzed for 13,371 SNPs, overlaps with the current study containing 444 neuroinvasive cases and 829 control patients, Table S3: Power calculations for detection of gene—gene interactions among the WNV dataset. Calculations were based on 444 severe disease cases and 829 non-severe infections, population prevalence of severe WNV of 0.01, and a range of minor allele frequencies (MAF). Each loci has log-additive inheritance and marginal odds ratio (OR) of 1.5, which reflects the top SNPs from the initial study. There is sufficient power (>0.80) to detect interaction ORs depicted in each cell or higher for the two corresponding MAF. Bonferroni-corrected two-sided *p*-value = 4 × 10^−6^, assuming 110 pairwise interactions.

## References

[1] World Health Organization (2017). Global Vector Control Response 2017–2030.

[2] World Health Organization West Nile Virus. http://www.who.int/news-room/fact-sheets/detail/west-nile-virus.

[3] GIDEON “West Nile Fever”—Staying in Real Time. https://www.gideononline.com/cases/westnilefever/.

[4] Centers for Disease Control and Prevention West Nile Virus. https://www.cdc.gov/westnile/index.html.

[5] Huhn G.D., Sejvar J.J., Montgomery S.P., Dworkin M.S. (2003). West Nile virus in the United States: An update on an emerging infectious disease. Am. Fam. Physician.

[6] Cahill M.E., Yao Y., Nock D., Armstrong P.M., Andreadis T.G., Diuk-Wasser M.A., Montgomery R.R. (2017). West Nile Virus Seroprevalence, Connecticut, USA, 2000–2014. Emerg. Infect. Dis..

[7] Montgomery R.R., Murray K.O. (2015). Risk factors for West Nile virus infection and disease in populations and individuals. Expert Rev. Anti Infect. Ther..

[8] Yeung M.W., Shing E., Nelder M., Sander B. (2017). Epidemiologic and clinical parameters of West Nile virus infections in humans: A scoping review. BMC Infect. Dis..

[9] Bai F., Thompson E.A., Vig P.J.S., Leis A.A. (2019). Current Understanding of West Nile Virus Clinical Manifestations, Immune Responses, Neuroinvasion, and Immunotherapeutic Implications. Pathogens.

[10] Cahill M.E., Conley S., DeWan A.T., Montgomery R.R. (2018). Identification of genetic variants associated with dengue or West Nile virus disease: A systematic review and meta-analysis. BMC Infect. Dis..

[11] Loeb M., Eskandarian S., Rupp M., Fishman N., Gasink L., Patterson J., Bramson J., Hudson T.J., Lemire M. (2011). Genetic variants and susceptibility to neurological complications following West Nile virus infection. J. Infect. Dis..

[12] Kenney A.D., Dowdle J.A., Bozzacco L., McMichael T.M., St Gelais C., Panfil A.R., Sun Y., Schlesinger L.S., Anderson M.Z., Green P.L. (2017). Human Genetic Determinants of Viral Diseases. Annu. Rev. Genet..

[13] Mozzi A., Pontremoli C., Sironi M. (2018). Genetic susceptibility to infectious diseases: Current status and future perspectives from genome-wide approaches. Infect. Genet. Evol..

[14] Gibson G. (2012). Rare and common variants: Twenty arguments. Nat. Rev. Genet..

[15] Ulbert S. (2019). West Nile virus vaccines-current situation and future directions. Hum. Vaccin. Immunother..

[16] Purcell S., Neale B., Todd-Brown K., Thomas L., Ferreira M.A., Bender D., Maller J., Sklar P., de Bakker P.I., Daly M.J. (2007). PLINK: A tool set for whole-genome association and population-based linkage analyses. Am. J. Hum. Genet..

[17] Kent W.J., Sugnet C.W., Furey T.S., Roskin K.M., Pringle T.H., Zahler A.M., Haussler D. (2002). The human genome browser at UCSC. Genome. Res..

[18] Howie B.N., Donnelly P., Marchini J. (2009). A flexible and accurate genotype imputation method for the next generation of genome-wide association studies. PLoS Genet..

[19] Delaneau O., Marchini J., Zagury J.F. (2011). A linear complexity phasing method for thousands of genomes. Nat. Methods.

[20] Auton A., Brooks L.D., Durbin R.M., Garrison E.P., Kang H.M., Korbel J.O., Marchini J.L., McCarthy S., McVean G.A., The 1000 Genomes Project Consortium (2015). A global reference for human genetic variation. Nature.

[21] Patterson N., Price A.L., Reich D. (2006). Population structure and eigenanalysis. PLoS Genet..

[22] Price A.L., Patterson N.J., Plenge R.M., Weinblatt M.E., Shadick N.A., Reich D. (2006). Principal components analysis corrects for stratification in genome-wide association studies. Nat. Genet..

[23] Risch N., Merikangas K. (1996). The future of genetic studies of complex human diseases. Science.

[24] Bonferroni C. (1935). Il Calcolo Delle Assicurazioni su Gruppi di Teste. Studi in Onore del Professore Salvatore Ortu Carboni.

[25] Turner S.D. (2014). qqman: An R package for visualizing GWAS results using Q-Q and manhattan plots. biorXiv.

[26] Gonzales T.K., Yonker J.A., Chang V., Roan C.L., Herd P., Atwood C.S. (2017). Myocardial infarction in the Wisconsin Longitudinal Study: The interaction among environmental, health, social, behavioural and genetic factors. BMJ Open.

[27] Herd P., Carr D., Roan C. (2014). Cohort profile: Wisconsin longitudinal study (WLS). Int. J. Epidemiol..

[28] Tryka K.A., Hao L., Sturcke A., Jin Y., Wang Z.Y., Ziyabari L., Lee M., Popova N., Sharopova N., Kimura M. (2014). NCBI’s Database of Genotypes and Phenotypes: dbGaP. Nucleic. Acids Res..

[29] Herd P. Wisconsin Longitudinal Study. https://www.ssc.wisc.edu/wlsresearch/.

[30] Wisconsin Longitudinal Study A Longitudinal Resource for Genetic Research in Behavioral and Health Sciences: Imputation Report, 1000 Genomes Project Reference Panel (Phase 3). https://www.ssc.wisc.edu/wlsresearch/documentation/GWAS/Herd_1000G_IMPUTE2report.pdf.

[31] Patterson N., Price A.L., Reich D. EIGENSOFT, 6.1.4. https://github.com/DReichLab/EIG.

[32] Centers for Disease Control and Prevention, National Center for Emerging and Zoonotic Infectious Diseases, Division of Vector-Borne Diseases Clinical Evaluation & Disease. https://www.cdc.gov/westnile/healthcareproviders/healthCareProviders-ClinLabEval.html.

[33] Lazear H.M., Diamond M.S. (2015). New insights into innate immune restriction of West Nile virus infection. Curr. Opin. Virol..

[34] Netland J., Bevan M.J. (2013). CD8 and CD4 T cells in west nile virus immunity and pathogenesis. Viruses.

[35] Glass W.G., McDermott D.H., Lim J.K., Lekhong S., Yu S.F., Frank W.A., Pape J., Cheshier R.C., Murphy P.M. (2006). CCR5 deficiency increases risk of symptomatic West Nile virus infection. J. Exp. Med..

[36] Bigham A.W., Buckingham K.J., Husain S., Emond M.J., Bofferding K.M., Gildersleeve H., Rutherford A., Astakhova N.M., Perelygin A.A., Busch M.P. (2011). Host genetic risk factors for West Nile virus infection and disease progression. PLoS ONE.

[37] Das R., Loughran K., Murchison C., Qian F., Leng L., Song Y., Montgomery R.R., Loeb M., Bucala R. (2016). Association between high expression macrophage migration inhibitory factor (MIF) alleles and West Nile virus encephalitis. Cytokine.

[38] Danial-Farran N., Eghbaria S., Schwartz N., Kra-Oz Z., Bisharat N. (2015). Genetic variants associated with susceptibility of Ashkenazi Jews to West Nile virus infection. Epidemiol. Infect..

[39] Lim J.K., Lisco A., McDermott D.H., Huynh L., Ward J.M., Johnson B., Johnson H., Pape J., Foster G.A., Krysztof D. (2009). Genetic variation in OAS1 is a risk factor for initial infection with West Nile virus in man. PLoS Pathog..

[40] Yakub I., Lillibridge K.M., Moran A., Gonzalez O.Y., Belmont J., Gibbs R.A., Tweardy D.J. (2005). Single nucleotide polymorphisms in genes for 2′-5′-oligoadenylate synthetase and RNase L inpatients hospitalized with West Nile virus infection. J. Infect. Dis..

[41] Van den Broeke C., Jacob T., Favoreel H.W. (2014). Rho’ing in and out of cells: Viral interactions with Rho GTPase signaling. Small GTPases.

[42] Fraisier C., Camoin L., Lim S.M., Bakli M., Belghazi M., Fourquet P., Granjeaud S., Osterhaus A.D., Koraka P., Martina B. (2013). Altered protein networks and cellular pathways in severe west nile disease in mice. PLoS ONE.

[43] Foo K.Y., Chee H.Y. (2015). Interaction between Flavivirus and Cytoskeleton during Virus Replication. Biomed Res. Int..

[44] Moni M.A., Lio P. (2017). Genetic Profiling and Comorbidities of Zika Infection. J. Infect. Dis..

[45] Hau P.M., Tsao S.W. (2017). Epstein-Barr Virus Hijacks DNA Damage Response Transducers to Orchestrate Its Life Cycle. Viruses.

[46] Dheekollu J., Lieberman P.M. (2011). The replisome pausing factor Timeless is required for episomal maintenance of latent Epstein-Barr virus. J. Virol..

[47] Qian F., Chung L., Zheng W., Bruno V., Alexander R.P., Wang Z., Wang X., Kurscheid S., Zhao H., Fikrig E. (2013). Identification of genes critical for resistance to infection by West Nile virus using RNA-Seq analysis. Viruses.

[48] Qian F., Goel G., Meng H., Wang X., You F., Devine L., Raddassi K., Garcia M.N., Murray K.O., Bolen C.R. (2015). Systems immunology reveals markers of susceptibility to West Nile virus infection. Clin. Vaccine Immunol..

[49] Qian F., Thakar J., Yuan X., Nolan M., Murray K.O., Lee W.T., Wong S.J., Meng H., Fikrig E., Kleinstein S.H. (2014). Immune markers associated with host susceptibility to infection with West Nile virus. Viral Immunol..

[50] McGuckin Wuertz K., Treuting P.M., Hemann E.A., Esser-Nobis K., Snyder A.G., Graham J.B., Daniels B.P., Wilkins C., Snyder J.M., Voss K.M. (2019). STING is required for host defense against neuropathological West Nile virus infection. PLoS Pathog..

[51] Zimmerman M.G., Bowen J.R., McDonald C.E., Pulendran B., Suthar M.S. (2019). West Nile Virus Infection Blocks Inflammatory Response and T Cell Costimulatory Capacity of Human Monocyte-Derived Dendritic Cells. J. Virol..

[52] Mitchell B.D., Fornage M., McArdle P.F., Cheng Y.C., Pulit S.L., Wong Q., Dave T., Williams S.R., Corriveau R., Gwinn K. (2014). Using previously genotyped controls in genome-wide association studies (GWAS): Application to the Stroke Genetics Network (SiGN). Front. Genet..

[53] Centers for Disease Control and Prevention, National Center for Emerging and Zoonotic Infectious Diseases, Division of Vector-Borne Diseases West Nile Virus Disease Cases and Deaths Reported to CDC by Year and Clinical Presentation, 1999–2018. https://www.cdc.gov/westnile/statsmaps/cumMapsData.html.

[54] Chancey C., Grinev A., Volkova E., Rios M. (2015). The global ecology and epidemiology of West Nile virus. Biomed Res. Int..

[55] Gene, National Center for Biotechnology Information, USA National Library of Medicine HS3ST4 Heparan Sulfate-Glucosamine 3-Sulfotransferase 4 [Homo Sapiens (Human)]. https://www.ncbi.nlm.nih.gov/gene?term=9951.

[56] Denys A., Allain F. (2019). The Emerging Roles of Heparan Sulfate 3-O-Sulfotransferases in Cancer. Front. Oncol..

[57] Biroccio A., Cherfils-Vicini J., Augereau A., Pinte S., Bauwens S., Ye J., Simonet T., Horard B., Jamet K., Cervera L. (2013). TRF2 inhibits a cell-extrinsic pathway through which natural killer cells eliminate cancer cells. Nat. Cell Biol..

[58] Yao Y., Strauss-Albee D.M., Zhou J.Q., Malawista A., Garcia M.N., Murray K.O., Blish C.A., Montgomery R.R. (2017). The natural killer cell response to West Nile virus in young and old individuals with or without a prior history of infection. PLoS ONE.

[59] Wang T., Welte T. (2013). Role of natural killer and Gamma-delta T cells in West Nile virus infection. Viruses.

[60] Luu P.-L., Ong P.-T., Dinh T.-P., Clark S.J. (2020). Benchmark study comparing liftover tools for genome conversion of epigenome sequencing data. NAR Genom. Bioinform..

[61] Pan B., Kusko R., Xiao W., Zheng Y., Liu Z., Xiao C., Sakkiah S., Guo W., Gong P., Zhang C. (2019). Correction to: Similarities and differences between variants called with human reference genome HG19 or HG38. BMC Bioinform..

[62] Browning B.L., Browning S.R. (2016). Genotype Imputation with Millions of Reference Samples. Am. J. Hum. Genet..

[63] Shi S., Yuan N., Yang M., Du Z., Wang J., Sheng X., Wu J., Xiao J. (2018). Comprehensive Assessment of Genotype Imputation Performance. Hum. Hered..

[64] Brinster R., Kottgen A., Tayo B.O., Schumacher M., Sekula P., Consortium C.K. (2018). Control procedures and estimators of the false discovery rate and their application in low-dimensional settings: An empirical investigation. BMC Bioinform..

[65] Fadista J., Manning A.K., Florez J.C., Groop L. (2016). The (in)famous GWAS P-value threshold revisited and updated for low-frequency variants. Eur. J. Hum. Genet..

[66] Panagiotou O.A., Ioannidis J.P., Genome-Wide Significance P. (2012). What should the genome-wide significance threshold be? Empirical replication of borderline genetic associations. Int. J. Epidemiol..

[67] Xu C., Tachmazidou I., Walter K., Ciampi A., Zeggini E., Greenwood C.M., Consortium U.K. (2014). Estimating genome-wide significance for whole-genome sequencing studies. Genet. Epidemiol..

[68] Haralambieva I.H., Ovsyannikova I.G., Pankratz V.S., Kennedy R.B., Jacobson R.M., Poland G.A. (2013). The genetic basis for interindividual immune response variation to measles vaccine: New understanding and new vaccine approaches. Expert Rev. Vaccines.

[69] Mentzer A.J., O’Connor D., Pollard A.J., Hill A.V. (2015). Searching for the human genetic factors standing in the way of universally effective vaccines. Philos. Trans. R. Soc. Lond. B Biol. Sci..

[70] Noll K.E., Whitmore A.C., West A., McCarthy M.K., Morrison C.R., Plante K.S., Hampton B.K., Kollmus H., Pilzner C., Leist S.R. (2020). Complex Genetic Architecture Underlies Regulation of Influenza-A-Virus-Specific Antibody Responses in the Collaborative Cross. Cell Rep..

[71] Johnson J.L. (2017). Genetic Association Study (GAS) Power Calculator.

[72] National Center for Biotechnology Information, USA. National Library of Medicine Wisconsin Longitudinal Study on Aging. dbGaP Study Accession: phs001157.v1.p1. https://www.ncbi.nlm.nih.gov/projects/gap/cgi-bin/study.cgi?study_id=phs001157.v1.p1.

