# Supplementary material for: In-Depth Analysis of Genetic Variation Associated with Severe West Nile Viral Disease
Source: Vaccines (Basel). 2020 Dec 8;8(4):744. doi: 10.3390/vaccines8040744 (PMC7768385; doi:10.3390/vaccines8040744)
Supplement: Supplementary file 1 [file vaccines-08-00744-s001.pdf]

Supplemental Materials

Figures and Tables

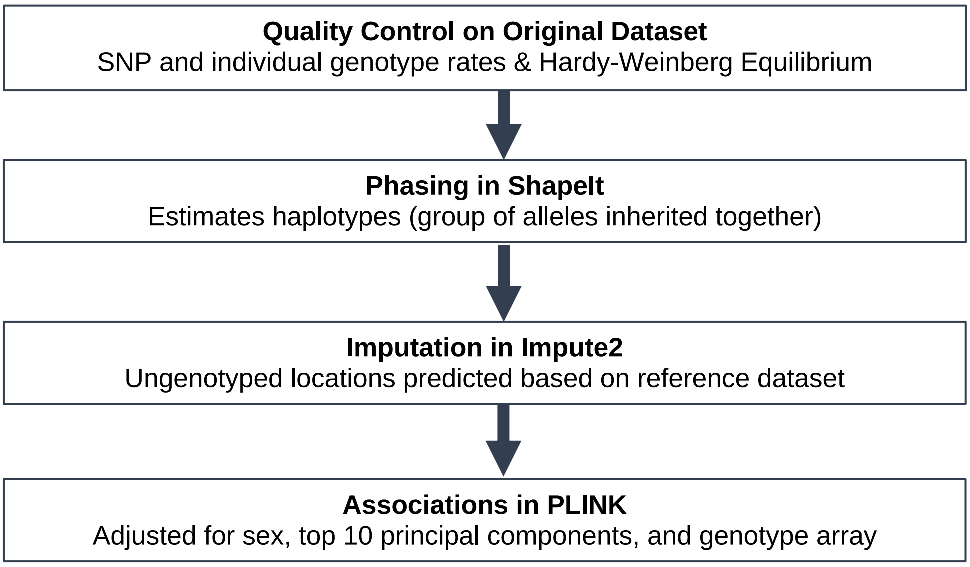

Supplemental Figure 1: Quality control and analysis flowchart. Overview of approach for quality control, phasing, imputation, and association analysis of the genetic dataset of WNV sample.

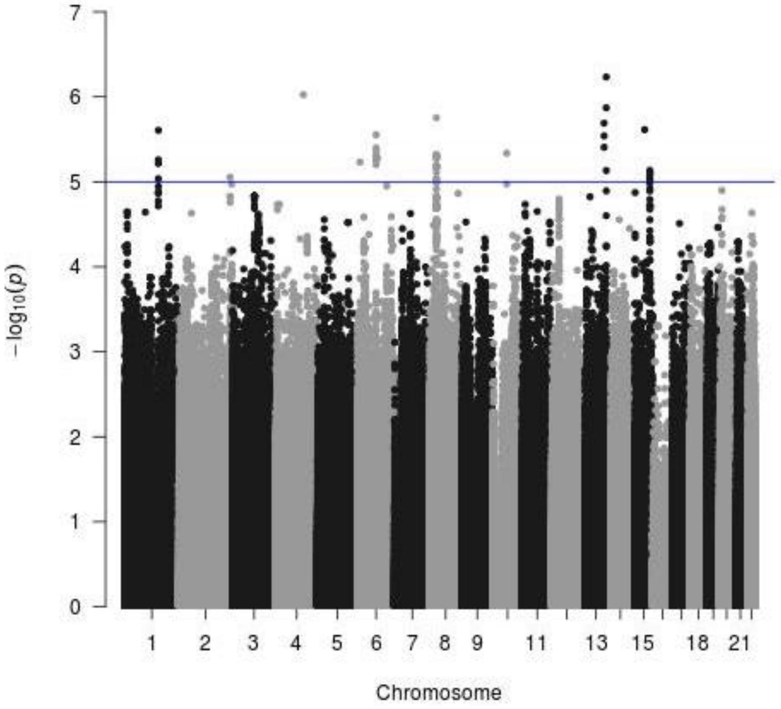

Supplemental Figure 2. Manhattan plot of imputed dataset of WNV severe cases and mild and asymptomatic infection controls. The chromosomes are along the x-axis and the negative log p-value along the y-axis. The blue line indicates SNPs that approach statistical significance, and the red line separate SNPs that reach the genome-wide corrected p-value threshold. The top association hits are located in a region of chromosome 13.

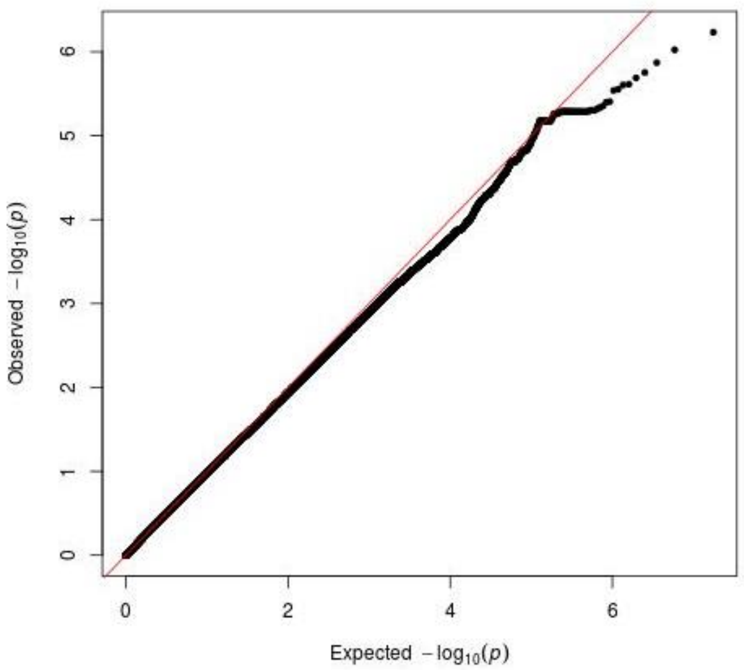

Supplemental Figure 3. QQ plot of the expected and observed negative log of the p-values from the association analysis of the imputed WNV dataset. The negative log of the observed p-values (x-axis) are plotted against the negative-log of the expected p-values (y-axis). The majority of the SNPs fall along the red line, indicating the results are not inflated.

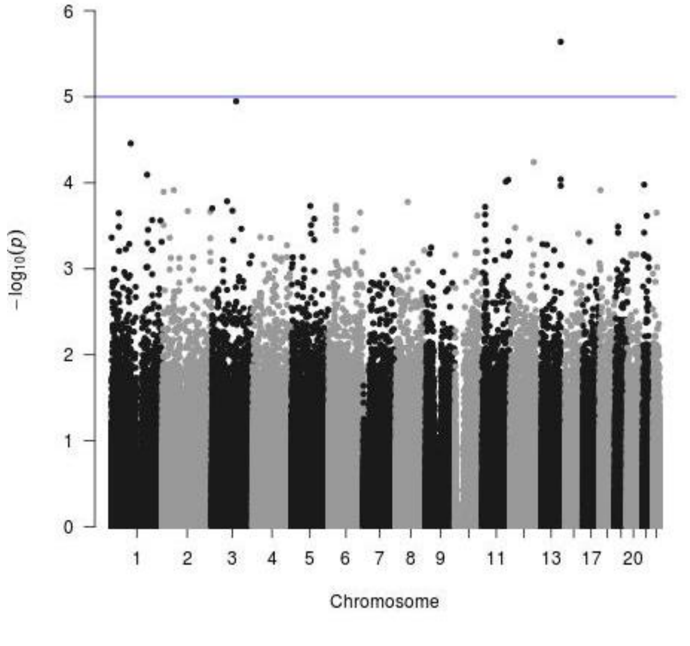

Supplemental Figure 4. Manhattan plot of imputed datasets of WNV severe cases, non-severe WNV-infected controls, and population controls from the Wisconsin Longitudinal Study on Aging. The chromosomes are along the x-axis and the negative log p-value along the y-axis. The blue line indicates SNPs that approach statistical significance, with none of the SNPs reaching statistical significance.

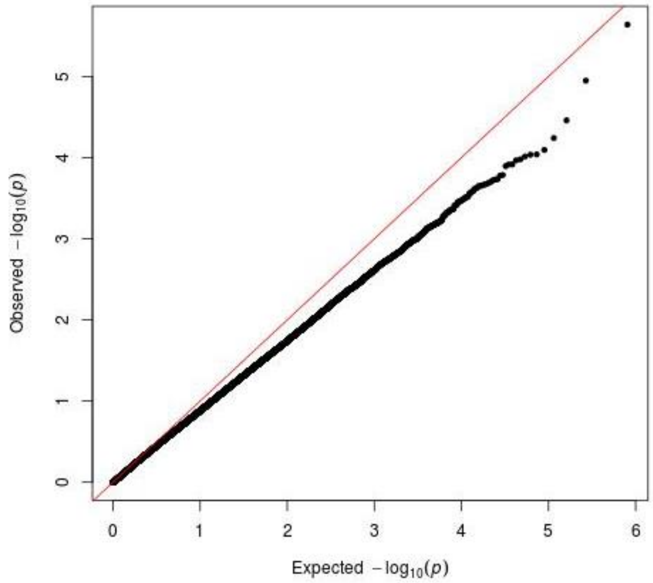

Supplemental Figure 5. QQ plot of the expected and observed negative log of the p-values from the association analysis of the imputed datasets of WNV severe cases, non-severe WNV-infected controls, and population controls from the Wisconsin Longitudinal Study on Aging. The negative log of the observed p-values (x-axis) are plotted against the negative-log of the expected p-values (y-axis).

Supplemental Table 1. Imputation of the West Nile virus dataset. The number of directly genotyped SNPs and the total number of SNPs following imputation, by chromosome. The 'before imputation' numbers only include directly genotyped SNPs; the 'after imputation' includes both directly genotyped and imputed SNPs.

Chromosome	SNPs before Imputation	SNPs after Imputation
1	122,328	951,641
2	117,487	1,037,275
3	96,489	876,211
4	85,854	890,168
5	87,466	780,119
6	100,143	838,789
7	77,796	575,678
8	74,558	673,065
9	63,680	532,225
10	73,544	453,872
11	73,458	609,238
12	71,897	593,833
13	51,389	447,677
14	46,154	398,047
15	43,006	345,655
16	45,934	352,000
17	42,821	326,054
18	39,878	337,128
19	33,751	271,962
20	36,933	267,593
21	19,672	161,495
22	22,300	164,147
**Total**	1,426,538	11,883,872

Supplemental Table 2. Comparison of p-values for the top three SNPs from the previously published GWAS within the current subset, with no covariate adjustment to reflect the original analysis. The previously published GWAS, including 560 neuroinvasive case patients and 950 control patients analyzed for 13,371 SNPs,[1] overlaps with the current study containing 444 neuroinvasive cases and 829 control patients.

CHR	SNP	Gene	Base Position	Previous Study P-value	Current Study P-value
4	rs2066786	RFC1	39302029	1.88×10^{-5}	4.98×10^{-4}
2	rs2298771	SCN1A	166892788	5.87×10^{-5}	5.56×10^{-3}
15	rs25651	ANPEP	90335788	1.44×10^{-4}	4.82×10^{-4}

Supplemental Table 3: Power calculations for detection of gene—gene interactions among the WNV dataset. Calculations were based on 444 severe disease cases and 829 non-severe infections, population prevalence of severe WNV of 0.01, and a range of minor allele frequencies (MAF). Each loci has log-additive inheritance and marginal odds ratio (OR) of 1.5, which reflects the top SNPs from the initial study. There is sufficient power (>0.80) to detect interaction ORs depicted in each cell or higher for the two corresponding MAF. Bonferroni-corrected two-sided p-value = 4×10^{-6}, assuming 110 pairwise interactions.

		**Minor Allele Frequency of SNP 1**		
		**0.15**	**0.25**	**0.35**
**Minor Allele Frequency of SNP 2**	**0.15**	2.95	2.64	2.57
	**0.25**		2.42	2.41
	**0.35**			2.47

Reference

1 Loeb, M. et al. Genetic variants and susceptibility to neurological complications following West Nile virus infection. *J Infect Dis* **204**, 1031-1037, doi:10.1093/infdis/jir493 (2011).
